# “Strengthening Dementia Care: Advancing Through ADI Accreditation for Excellence and Acknowledgment”

**DOI:** 10.1002/alz.084921

**Published:** 2025-01-09

**Authors:** Amalia Fonk Utomo, Diego Aguilar

**Affiliations:** ^1^ Alzheimer's Disease International, London United Kingdom; ^2^ Alzheimer’s Disease International, London United Kingdom

## Abstract

**Background:**

The ADI Accreditation Programme, launched in December 2020, aims to support all Alzheimer and dementia associations and other organizations, in improving care for people living with dementia. By providing a standards‐based approach to knowledge and skills, ADI establishes benchmarks that program providers must adhere to. Successful completion of an evaluation allows carers, trainers & program providers to earn ADI Accreditation, indicating that they have reached the required global standard for their training and learning activities, including culturally appropriate context to improve care quality. This program is open to ADI members, and organizations including universities & training colleges.

**Activities:**

Our Global Review Panel (GRP) Members consists of multi‐discipline, cultural and regional experts in the global dementia field. Successful completion of an evaluation process: initial application, evaluation report, virtual/in‐person or hybrid visit from our GRP Members, the program/providers can earn it.

The first provider accredited was Kiang Wu Nursing College (KWNC) of Macau on January 25, 2021. The project involved discussions on external‐internal governance, trainers‐program committees, students, graduates & other stakeholders. A 3‐day virtual visit to KWNC in September, including evaluation, involved carers, trainers, students & collaboration with other organizations and people living with dementia, followed by a final review by the GRP in October‐November.

Silverado Memory Care became the first program to be accredited for their Dementia Care Program on December 22, 2022. In‐person visits took place on August 16‐17 2022. University of Bradford UK, became the first university in Europe to be accredited their Centre for Dementia Applied Science on December 22, 2022. In‐person visit took place on June 20‐21 June 2023. Wicking Dementia Research & Education Centre University of Tasmania, received their accreditation after virtual visit on 29‐30 August 2023. All process involving trainers, stakeholders, associates & staff in 2024 Dementia Australia is currently on the process along with new potentials.

**Results:**

KWNC Macau, Silverado, University of Bradford & University of Tasmania received ADI Accreditation. Throughout the program, there has been an increase in awareness and understanding of the importance of high‐quality culturally contextualized carer training achieved.

